# Nanoparticle Drug Delivery Systems Designed to Improve Cancer Vaccines and Immunotherapy

**DOI:** 10.3390/vaccines3030662

**Published:** 2015-08-27

**Authors:** Yuchen Fan, James J. Moon

**Affiliations:** 1 Department of Pharmaceutical Sciences, University of Michigan, Ann Arbor, MI 48109, USA; E-Mail: yuchfan@umich.edu; 2 Biointerfaces Institute, University of Michigan, Ann Arbor, MI 48109, USA; 3 Department of Biomedical Engineering, University of Michigan, Ann Arbor, MI 48109, USA

**Keywords:** cancer immunotherapy, nanotechnology, cancer vaccine, lymphoid draining, adjuvant, dendritic cell, immune checkpoint, adoptive cell therapy

## Abstract

Recent studies have demonstrated great therapeutic potential of educating and unleashing our own immune system for cancer treatment. However, there are still major challenges in cancer immunotherapy, including poor immunogenicity of cancer vaccines, off-target side effects of immunotherapeutics, as well as suboptimal outcomes of adoptive T cell transfer-based therapies. Nanomaterials with defined physico-biochemical properties are versatile drug delivery platforms that may address these key technical challenges facing cancer vaccines and immunotherapy. Nanoparticle systems have been shown to improve targeted delivery of tumor antigens and therapeutics against immune checkpoint molecules, amplify immune activation via the use of new stimuli-responsive or immunostimulatory materials, and augment the efficacy of adoptive cell therapies. Here, we review the current state-of-the-art in nanoparticle-based strategies designed to potentiate cancer immunotherapies, including cancer vaccines with subunit antigens (e.g., oncoproteins, mutated neo-antigens, DNA and mRNA antigens) and whole-cell tumor antigens, dendritic cell-based vaccines, artificial antigen-presenting cells, and immunotherapeutics based on immunogenic cell death, immune checkpoint blockade, and adoptive T-cell therapy.

## 1. Introduction

Immunotherapy has been explored for more than a century as a potential therapeutic approach to combat against cancer. Dating back to 1891 when neither chemotherapy nor radiotherapy were developed, a surgeon named William B. Coley successfully treated his cancer patients with bacterial products, which are now recognized to have induced non-specific anti-tumor inflammation [1]. However, immunotherapeutic strategies for cancer treatment have been doubted for a long time due to disappointing failures in various clinical trials. It was not until very recent years that dendritic cell-based vaccines and immune checkpoint inhibitors have each ushered new line of cancer therapy and raised the hope for unleashing patients’ own immune system to eradicate tumors [2,3]. Cancer immunity consists of several key steps, including release of antigens from tumor beds, presentation of tumor antigens by antigen-presenting cells (APCs), priming and activation of T cells by activated APCs, migration and infiltration of effector T cells back to the tumor, and finally the recognition and killing of tumor cells by effector T cells [4]. In theory, each of these steps can be targeted with various therapeutic approaches. The current advancement in cancer immunotherapy is mainly driven by striking results obtained with inhibitors of negative immune checkpoint molecules. However, this approach is mostly aimed at augmenting the potency of pre-existing tumor-specific T cells and benefits only a portion of patients as seen in recent clinical trials [5,6]. In contrast, cancer vaccines targeting early steps of antigen processing can potentially improve both therapeutic and prophylactic efficacies against not only primary tumor but also inoperable metastasis or relapse, and benefit more patients, especially those that lack sufficient levels of pre-existing anti-tumor T cells and/or immune checkpoint molecules.

**Figure 1:**
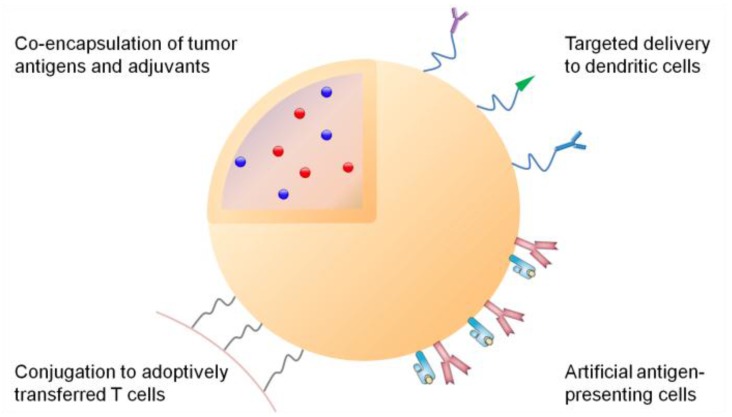
Multi-functional nanoparticles for cancer immunotherapy. Tumor antigens and adjuvants can be co-loaded into the particle core, while the particle surface can be modified with antibodies or ligands specific to dendritic cells; or major histocompatibility complex/antigen complexes and co-stimulatory ligands as artificial antigen-presenting cells. Additionally, nanoparticles loaded with immune potentiators can be conjugated on T cells to improve adoptive T cell therapy.

However, despite tremendous potential of cancer vaccines, successful treatment and eradication of tumors with cancer vaccines has been elusive due to insufficient induction of immune responses with conventional vaccination approaches [7]. This highlights the need for new vaccination strategies that can efficiently deliver tumor antigens and adjuvants to APCs and stimulate immune responses strong enough to kill tumor cells. In this regard, nanoparticles have been intensively investigated for over the past three decades as delivery vehicles of traditional chemotherapeutics targeted to solid tumors [8,9,10,11,12,13]. Repurposing these nanomaterials to target the immune system may offer new opportunities to tune immunity and elicit strong anti-tumoral immune responses [14,15,16]. Indeed, multi-functional nanomaterials have several key advantages over conventional therapeutics for cancer immunotherapy (Figure 1): (1) nanoparticles carrying both tumor antigens and adjuvants can stably co-deliver vaccine components to APCs [17,18]; (2) nanocarriers with finely tuned size and a defined surface chemistry can achieve selective delivery to lymphoid tissues [19,20,21], while nanoparticles composed of biomaterials with immune-stimulating properties may also serve a dual role as a vaccine carrier and an adjuvant, thus simplifying the vaccine design [22,23]; (3) surfaces of nanomaterials can also be engineered to display antigens and co-stimulatory ligands to serve as artificial APC (aAPC) and potentiate T cell immune responses [24,25]; (4) delivery systems designed to initiate immunogenic cell death or target immune checkpoint molecules can drive anti-tumoral immune responses and reverse immune suppression; and (5) therapeutics-loaded particles can be utilized to improve anti-tumoral efficacy of adoptive T cell therapy [26]. This review article covers exciting new developments in each of these key areas of research, highlighting the potential of nanoparticle-based immunotherapy against cancer.

## 2. Principles of Adaptive Immunity against Cancer

Vertebrates are protected by the immune system from pathogens such as viruses, bacteria, fungi and parasites. Immune responses to foreign pathogens can be classified into two categories, namely innate and adaptive immunity. Innate immunity provides rapid defense against pathogens while adaptive immunity requires processing of pathogens by APCs, presentation of immunogenic antigens to T cells and B cells, and elicitation of cellular and humoral immune responses. APCs play a pivotal role at the interface of innate and adaptive immune responses. Professional APCs include B cells, macrophages, and dendritic cells (DCs), among which DCs have been considered as the most efficient APC population [27]. DCs can process endogenous or exogenous antigens in the context of major histocompatibility complex (MHC) class I or II, and present the MHC/antigen peptide complex as the activation “signal 1” to CD8^+^ and CD4^+^ T cells, respectively. Activation of T cells requires an additional “signal 2” induced by ligation of co-stimulatory markers CD80/86 on DCs with CD28 on T cells, as well as a T-cell polarizing “signal 3” provided by cytokines secreted by DCs. Although MHC-I is constitutively expressed by the majority of mammalian cells, non-professional APCs cannot provide “signal 2 and 3” to alert the immune system when infected with pathogens. Therefore, antigen processing and presentation by APCs are a crucial first step in initiation of adaptive immune responses. In particular, DCs have a unique ability to process exogenous pathogens and activate CD8^+^ T cells via a process known as cross-presentation. Although the exact mechanisms of cross-presentation are still under investigation [28,29], vacuolar and cytosolic pathways have been identified [30]. The main difference between the two lies in the intracellular location for processing and loading of antigens to MHC-I: the vacuolar pathway utilizes endosomes while the cytosolic pathway utilizes endoplasmic reticulum for formation of MHC-I/antigen peptide complexes. Notably, increase in endosomal pH is thought to prevent excessive protease-mediated degradation of antigens in endosomes, thus promoting cross-presentation [31]. In addition, certain DC subsets such as tissue-resident CD8^+^ and migratory CD103^+^ DCs are known to be more efficient at antigen cross-presentation than other DC subtypes in mice [32], and human CD141^+^/BDCA-3^+^ DCs have been recently proposed as the functional equivalent of the murine CD8^+^ DCs [33].

Once activated in lymphoid tissues, cytotoxic CD8^+^ T lymphocytes (CTLs) enter the systemic circulation and patrol peripheral tissues in search of target cells. When CTLs identify target cells displaying a specific antigen epitope in the context of MHC-I, they secrete perforin and granzymes to lyse the target cells, and within minutes they move on to kill the next target [34]. CD4^+^ T cells mainly play an indirect/helper role. Following activation by MHC-II/antigen peptide complex presented by DCs, naïve CD4^+^ T cells differentiate into distinctive subtypes of helper T (T_H_) cells depending on the polarizing cytokines [35]. The T_H_1 population induced by IL-12 secretes IL-2 and IFN-γ and drives CD8^+^ T cell responses while T_H_2 and regulatory T cells (T_reg__s_) induced by IL-4 and TGF-β are involved in humoral immune responses and immune suppression, respectively. In addition, CD4^+^ helper T cells express CD40L, which feeds back to DCs to further amplify immune activation and aid in establishment of memory CD8^+^ T cell responses [36,37].

In theory, immune system can inhibit oncogenesis by actively identifying and eliminating cancerous cells, a process referred to as immunosurveillance [38]. However, tumor cells have devised mechanisms to evade immune responses, including down-regulation of tumor antigens and promotion of immunosuppression [39,40]. Established tumor microenvironment is generally immunosuppressive due to up-regulation of inhibitory molecules against T cells. Activated T cells up-regulate cytotoxic T-lymphocyte-associated protein 4 (CTLA-4) that binds to co-stimulatory molecules on DCs with higher affinity than CD28. Although CTLA-4 naturally serves as a peripheral inhibitory signal to prevent over-reactivity of T cells, it also dampens anti-tumor immune responses. Besides, subsets of tumor cells highly express programmed death-ligand 1 (PD-L1) that binds to programmed death-1 (PD-1) on T cells and inhibit their effector functions [40]. Tumor cells can also secrete cytokines such as IL-10 and TGF-β, which both directly inhibit the proliferation of CTLs and drive the differentiation of T_regs_ that provide an additional source of these immunosuppressive cytokines. In addition to T_regs_, tumor cells can recruit other inhibitory immune cells such as macrophages and myeloid-derived suppressor cells (MDSCs) to further dampen cytotoxic functions of CTLs [41]. Thus, tumor cells can promote immunosuppressive tumor microenvironment and shield themselves from CTLs by hijacking normal negative feedback loops designed to guard against excessive activation of T cell responses.

## 3. Synthetic Systems for Delivery of Tumor Antigens

Tumor antigens can be categorized broadly into subunit antigens and whole-cell antigens. Subunit antigens include altered cell-surface polysaccharides, peptides, oncoproteins, and DNA and mRNA that encode those proteins, while tumor-cell lysate and immunogenically dying tumor cells can serve as the source of whole-cell antigens. Table 1 presents major advantages and challenges for each class of tumor antigens utilized for cancer vaccination. Some viruses (e.g., Epstein-Barr virus (EBV), human papilloma virus (HPV) and hepatitis B and C viruses) contribute to cancer development, and virally encoded gene products can also serve as the potential targets of immunotherapy [42]. Among different types of tumor antigens, oncoproteins, which are either mutated or over-expressed normal or embryonic proteins from fetal development, are intensively investigated for cancer vaccines due to their potential to elicit broad-epitope CD8^+^ and CD4^+^ T-cell responses. In comparison to full-length protein antigens that require cellular uptake and processing, peptide epitopes can directly bind to MHC molecules, and their stability is less affected during the preparation and storage of vaccine products. In line with these advantages, there are many ongoing clinical trials on peptide-based cancer vaccines [43]. However, the major challenge facing cancer vaccination based on subunit antigens is their poor immunogenicity and limited therapeutic efficacy. For example, in the case of melanoma-associated antigens, β-catenin, survivin, tyrosinase, gp100, MAGE, melan-A (MART1), and NY-ESO-1, have been identified and tested in clinical trials [44]. In a Phase III trial of gp100 peptide in combination with IL-2 and Montanide^TM^ ISA 51 as adjuvants, the response rate and overall survival improved from 6% to 16% and 11.1 months to 17.8 month, respectively, in comparison to the IL-2 alone treatment group [45]. However, another Phase III trial with MAGE-A3 peptide has failed to prolong disease-free survival [44]. Overall, therapeutic efficacy of cancer vaccines remain suboptimal, partially due to the fact that many tumor antigens evaluated in clinical trials are self-antigens against which auto-reactive T-cells are eliminated or tolerized. In addition, conventional vaccine/adjuvant delivery systems have limited capability to target delivery of tumor antigens and adjuvants to proper APCs and intracellular compartments. In this regard, nanotechnology-based vaccine systems are poised to address these challenges as described below.

**Table 1:** Major advantages and remaining challenges for tumor antigens.

Tumor antigens		Advantages	Challenges
Subunit antigens	Polysaccharides	Defined chemical synthesis	Elicitation of humoral rather than cellular immune responses
	Peptides	Ease of production	Poor delivery efficiency
		Stable vaccine formulations	Monovalent immune response
		May not require antigen-processing by APCs	Subject to HLA-specificity
	Proteins	Broad-epitope immune responses Wide HLA-specificity	Poor delivery efficiency
			Suboptimal for CD8^+^ T cell responses
			Weak immunogenicity of self-antigens
	DNA and mRNA	Ease of production	Poor delivery efficiency
		*In situ* expression of full-length antigens	Poor *in vivo* stability
		Flexible to encode immune stimulators	Limited transfection efficiency
Whole-cell antigens	Tumor-cell lysate	Broad-epitope immune responses Potential for “personalized” therapy	Requires tissue biopsy
			Manufacturing challenges
			Loss of antigenicity during production
			Presence of self-antigens
	Immunogenically dying tumor cells	Broad-epitope immune responses	Requires additional therapeutic interventions Presence of self-antigens and immunosuppressive molecules, e.g., PD-L1
		Full preservation of tumor antigens	
		Potential for “personalized” therapy	

### 3.1. Efficient Draining to Lymphoid Tissues

**Figure 2:**
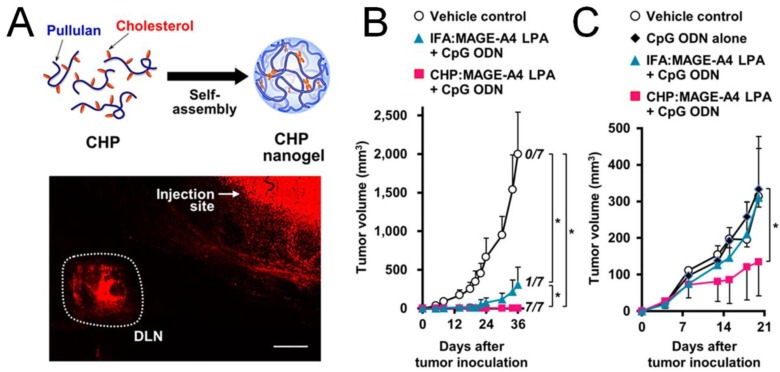
Anti-tumor efficacy improved by efficient lymphoid draining and retention of nanoparticle-based cancer vaccines. (**A**) Lymphoid draining of a fluorophore-labeled pullulan nanogel 6 h post subcutaneous injection to mice. Scale bar, 1 mm. The nanogel loaded with a long peptide antigen (LPA) MAGE-A4 achieved better prophylactic (**B**) and therapeutic (**C**) efficacy compared to the soluble antigen; (**B**) Mice were immunized on day -7, followed by inoculation of tumor on day 0; (**C**) mice were inoculated with tumor on day 0, followed by immunization on day 4 and 11. Reproduced with permission [47].

Nanocarriers can improve the efficacy of subunit cancer vaccines by facilitating antigen presentation and T-cell activation. This is achieved by exploiting efficient draining of nanocarriers to lymphoid tissues and their prolonged tissue residence as well as controlled release of antigens and adjuvants. Particle size is one of the primary factors determining the efficiency of lymphatic draining. Large particles (>500 nm in diameter) can be physically trapped at the injection site by interaction with extracellular matrix proteins, whereas ultra-small nanoparticles (<10 nm in diameter) or soluble antigen molecules can rapidly diffuse into and out of lymph nodes, thus minimizing the chance of APCs phagocytizing sufficient amount of vaccine particles [15]. On the other hand, particles of an intermediate size (10–100 nm in diameter) can both efficiently drain to regional draining lymph nodes and become retained there, thereby increasing the chance of antigen uptake and presentation by APCs [15,20]. Indeed, one study has compared the immunogenicity of protein or peptide antigen-conjugated nanobeads with sizes ranging from 0.02 to 2 μm. Following intradermal injection of these nanobeads into mice, a 40 nm nanovaccine formulation drained most efficiently to lymph nodes and elicited stronger antigen-specific T-cell immune responses than other formulations, including vaccines with conventional adjuvants such as Alum, monophosphoryl lipid A (MPLA), or Quil-A [46]. In another study, following subcutaneous injection into mice, nanogels with a mean diameter of 60 nm self-assembled by polysaccharide cholesteryl pullulan delivered synthetic long tumor-antigen peptides to medullary macrophages, which primed anti-tumoral CD8^+^ T cell responses in prophylactic and therapeutic settings (Figure 2) [47]. Furthermore, enhanced cytotoxic CD8^+^ T-cell responses and inhibition of tumor growth have been achieved by targeting nanovaccines to tumor-draining lymph nodes rather than non-tumor draining lymph nodes, suggesting that antigen-primed but immune-suppressed lymphoid tissues can serve as ideal sites of immune activation [48]. These studies rely on various imaging techniques with nanoparticles labeled with fluorescent dyes or contrast agents to track and quantify lymphatic draining. For example, a polyester nanoparticle system loaded with ovalbumin (OVA) and labeled with a near-infrared probe has been utilized to demonstrate co-transport of the antigen and nanoparticles to draining lymph nodes [49]. In an alternative approach, poly(lactic-co-glycolic acid) (PLGA) nanoparticles designed to carry iron oxide particles conjugated with fluorophore-labeled peptide antigen permitted bimodal tracking of the nanocarriers with MRI and fluorescent imaging [50]. Notably, it remains to be seen whether these delivery systems can be successfully translated into clinics since most studies were performed on murine models. Indeed, upon subdermal injection into the breast region of breast cancer patients, large (>300 nm) radio-labeled colloids were drained slowly through the lymph vessels and retained longer in sentinel lymph nodes, compared with their small (<50 nm) counterparts that rapidly visualized lymphatic vessels, sentinel lymph nodes, and second- and third-tier lymph nodes [51]. In addition, lymphatic draining of particulate vaccines also depends on the material composition, morphology, and surface chemistry of particles [21].

### 3.2. Targeted Delivery to Dendritic Cells

Once in contact with immune cells, tumor antigens have to be engulfed and processed by APCs, preferably DCs, to activate adaptive immune responses. Therefore, vaccine delivery targeted to DCs may be beneficial. Indeed, a protein vaccine composed of the full-length NY-ESO-1 fused to human mAb specific to DEC-205 (CD205), which is a C-type lectin receptor expressed on DCs, has been shown to elicit robust antigen-specific humoral and cellular immune responses with a good safety profile in a recent Phase I clinical trial [52]. Nanoparticles encapsulating tumor antigens can also be modified with targeting moieties on their surfaces to achieve DC-specific delivery. Such delivery systems specifically targeted to DEC-205, DC-SIGN, mannose receptor, Fc receptor, CD40, or CD11c have been reported [53,54,55]. Although these systems have been shown to induce stronger DC activation, compared with their non-targeted counterparts, it remains to be determined whether a particular targeting ligand is optimal for the DC-targeting approach. A recent study has addressed this issue by comparing PLGA nanovaccines modified with antibodies against CD40, DEC-205, or CD11c, and showed that the CD40 Ab-modified nanoparticles achieved the greatest binding to and uptake by DCs [56]. Interestingly, despite differential levels of DC-targeting, these various nanovaccine formulations induced similar levels of CD8^+^ T cell responses [56]. It should be noted that the extent of DC-targeting, particle uptake, and subsequent immune activation depends on specific physico-chemical properties of the nanocarrier itself as well as adjuvants employed in the vaccine system. Thus, efficiencies of DC-targeting and induction of adaptive immune responses by nanovaccines may have to be optimized in the context of each vaccine carrier and adjuvant. In particular, different DC subsets have distinctive sites of tissue residence, receptor expression profiles, and functions [57], and nanovaccines designed to target DC subsets with high efficiency of antigen cross-presentation, such as murine lymphoid tissue-resident CD8^+^ DCs and human CD141^+^/BDCA-3^+^ DCs and Langerhans cells [32,33], should be explored further.

### 3.3. Promotion of Cross-Presentation

Extracellular antigens are usually processed and presented via MHC-II by APCs to CD4^+^ T cells; however, tumor antigens engulfed by APCs need to be presented via MHC-I to activate CTLs, which are the main effector cells against tumor cells. Thus, traditional vaccine approaches relying on soluble protein or peptide tumor antigens may skew immune responses to CD4^+^ T cell responses, while failing to induce sufficient CTL responses. In contrast, tumor antigens delivered by functional nanomaterials designed to promote endosomal escape (*i.e.*, translocation of antigens from endosomes/phagosomes to cytosol) may induce cross-presentation and favorably elicit CD8^+^ T cell responses [32]. To this end, extensive research efforts have been focused on pH-sensitive delivery systems that can retain their cargo under the physiological pH condition while triggering release of antigens and disruption of endocytic vacuoles at the acidic (~pH 6) endosomal microenvironment [58]. For example, a liposomal antigen delivery system modified with a pH-sensitive dextran derivative has been shown to promote cytosolic delivery of antigens [59]. In addition, a micellar system composed of an amphiphilic polymer with a pH-sensitive building block forming the particle core has been devised to induce fusion of the nanomaterials to endosomal vesicles, thus transporting protein antigen surface-displayed on micelles from endosome to cytosol and promoting antigen cross-presentation and CD8^+^ T cell responses [60]. An alternative approach includes an oxidation-sensitive polymersome that can respond to the oxidative environment of endosomes and deliver antigens and adjuvants to cytosol [61]. Additionally, liposomes modified with a cell-penetrating peptide octaarginine or gold nanoparticles displaying tumor antigens were also shown to promote cross-presentation [62,63].

### 3.4. Co-Delivery of Adjuvants

**Figure 3:**
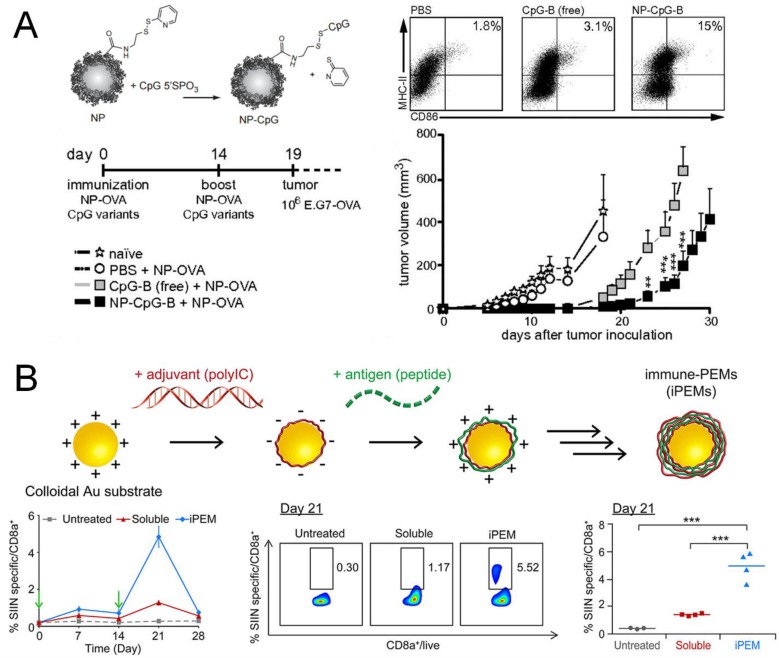
Co-delivery of antigens and adjuvants by nanoparticles. (**A**) TLR9 agonist CpG was conjugated on the surface of polymeric nanoparticles via disulfide exchange. The particulate adjuvant improved DC activation *in vitro* as well as prophylactic efficacy against tumor *in vivo*, compared with the soluble CpG; (**B**) TLR3 agonist poly I:C and an antigen peptide were complexed onto gold nanoparticles via electrostatic interactions, and elicited more antigen-specific CD8^+^ T cells compared with the soluble vaccine. Panel (**A**) reproduced with permission [68]; Panel (**B**) reproduced with permission [70].

Another major advantage of nanoparticle delivery systems lies in their ability to co-deliver antigens together with adjuvants, thereby enhancing cross-presentation and/or skewing immune responses to desired CD4^+^ T helper phenotypes. Agonists for Toll-like receptors (TLRs) have been widely investigated as adjuvants for cancer vaccines [64]. Although TLRs are mainly involved in innate immunity by sensing pathogenic danger signals, they are crucial for induction of adaptive immune responses as they can promote cross-presentation in APCs to activate CD8^+^ T cells or prime APCs to release cytokines that can polarize CD4^+^ T_H_ cells to specific phenotypes [65]. Since the T_H_1 responses elicited by activation of TLR3, TLR7, or TLR9 contribute to CD8^+^ T cell responses [66,67], agonists of these TLRs have been widely examined for cancer nanovaccines. CpG, which is an unmethylated oligonucleotide containing CpG motif, is a potent TLR9 agonist. CpG has been complexed with cationic polymers via the electrostatic interaction or conjugated with nanocarriers, which improved immune activation compared with administration of free soluble adjuvant (Figure 3A) [68,69]. The charge-mediated entrapment was also exploited to co-load an anionic TLR3 agonist poly I:C and cationic antigen peptides onto gold nanoparticles via the “layer-by-layer” strategy, leading to elicitation of robust antigen-specific CD8^+^ T cells when tested with a model antigen *in vivo* (Figure 3B) [70]. In addition to efficient loading of adjuvants, co-entrapment of an antigen and adjuvant within the same particles can also enhance the efficiency of cross-presentation and induction of CD8^+^ T cells, compared with soluble vaccine components admixed together [69,70,71]. Moreover, nanoparticles designed for multifaceted drug loading can support a combinational use of adjuvants, thus permitting exploitation of synergy among certain TLR agonists [72]. For example, CpG and poly I:C have been co-loaded into polyester nanoparticles [73], while the TLR4 agonist glucopyranosyl lipid A and TLR7 agonist imiquimod have been co-encapsulated into liposomes [74]. In both cases, the T_H_1 response was significantly improved by the dual TLR agonists-loaded particles, compared with that elicited by a single adjuvant. Alternatively, TLR agonists can be combined with siRNAs inhibiting the immunosuppressive pathways. Co-delivery of CpG and siRNA targeting IL-10, the inducer of T_H_2 and T_reg_ cells, skewed immune responses to the T_H_1 type [75]. The combination of peptide epitope of tyrosine-related protein 2 (Trp2) and CpG-based nanovaccine with siRNA against TGF-β, which is one of the major cytokines responsible for induction and maintenance of immunosuppressive tumor microenvironment, has significantly improved the therapeutic efficacy of nanoparticle-based cancer vaccine in a late-stage murine melanoma model [76].

### 3.5. Delivery of DNA and mRNA Tumor Antigens

DNA and mRNA encoding oncogenic proteins or peptides are appealing tumor antigens due to the ease of manufacturing scale-up and their potential for further modification with nucleic acid sequences that encode for proteins with immunostimulatory functions (e.g., flagellin). However, previous clinical trials on DNA cancer vaccines, majority of which were administered as naked DNA via the intramuscular route, showed generally poor response rates [77]. Although viral vectors and electroporation have been employed to improve the transfection of DNA vaccines, they are subject to safety and compliance issues [78]. Alternatively, synthetic delivery systems can be used to deliver DNA and mRNA therapeutics due to several advantages: (1) synthetic materials such as cationic lipids and polymers are safer alternatives to viral vectors; (2) gene therapeutics can be stabilized and protected from nuclease-mediated degradation by particulate carriers, and DNA and RNA have also been designed to self-assemble into distinctive nanostructures with improved colloidal stability [79,80]; (3) injection-free gene delivery routes can be exploited with DNA- and RNA-loaded nanocarriers, such as microneedles, pH-sensitive polymeric nanoparticles, or lipoplexes for non-parenteral routes of delivery [81,82,83]; and (4) nanocarriers can be modified with targeting moieties, e.g., mannose, to achieve DC-targeted delivery and transfection [84].

### 3.6. Delivery of Whole-Cell Cancer Vaccines

Compared with a single peptide or protein antigen, whole-cell cancer vaccines may elicit multivalent immune responses by broadening epitope recognition and help to realize personalized immunotherapy. Whole-cell antigen can be obtained from tumor cell lysates with necrotic features or inactivated whole tumor cells with apoptotic features. Similar to subunit vaccine nanocarriers, tumor cell lysates and TLR agonists have been co-encapsulated into particulate delivery systems, including liposomes or PLGA micro/nanoparticles [85,86]. Whole-cell cancer vaccine has also been delivered by a biodegradable, “infection-mimicking” PLGA matrix containing tumor lysate as the source of tumor antigens, granulocyte macrophage colony-stimulating factor (GM-CSF) for recruitment of DCs *in situ*, and CpG for activation of recruited DCs [87]. This PLGA matrix-based whole-cell cancer vaccine successfully elicited antigen-specific CD8^+^ T cells and improved both prophylactic and therapeutic anti-tumor efficacy, compared with a conventional whole-cell vaccine GVAX, composed of irradiated, GM-CSF-secreting tumor cells. In an alternative approach, plasma membrane of tumor cells has been extracted and coated onto polymeric nanoparticle cores along with the TLR4 agonist MPLA as a tumor cell-mimicking cancer vaccine [88].

## 4. Synthetic Delivery Systems for DC-Based Cancer Vaccines

Direct administration of autologous DCs activated by tumor antigens *ex vivo* may be an efficient approach for vaccination against cancers, as exemplified by the first and only approved therapeutic DC-based cancer vaccine, Sipuleucel-T (Provenge), which has improved the median survival of patients with metastatic castrate-resistant prostate cancer by 4.1 months, compared with the placebo group [89]. As introduced earlier, delivery of tumor antigens by particulate systems has been shown to enhance antigen processing and presentation by DCs; therefore, particle-based vaccine delivery applied to DC-based vaccines may improve their anti-tumoral efficacy. One study has employed antigen-loaded poly(γ-glutamic acid) nanoparticles to show that DCs activated by these particles released T_H_1 cytokines, elicited robust T-cell activation *in vitro,* and enhanced protection against tumor challenge in mice [90]. In another approach, antigen delivery by porous silica particles induced secretion of type I IFN cytokine from DCs, leading to reduced tumor growth in both therapeutic and prophylactic conditions [91]. The benefits of multi-drug loading within nanoparticles were also demonstrated in a TriMix delivery system [92]: the mixture of antigen and adjuvant mRNAs was encapsulated in cationic liposomes which were then conjugated to microbubbles to allow ultrasound-triggered transfection of DCs. DCs activated by this strategy exhibited enhanced therapeutic efficacy against established tumors, when compared with DCs primed with antigen mRNA with or without lipopolysaccharide (LPS).

**Figure 4:**
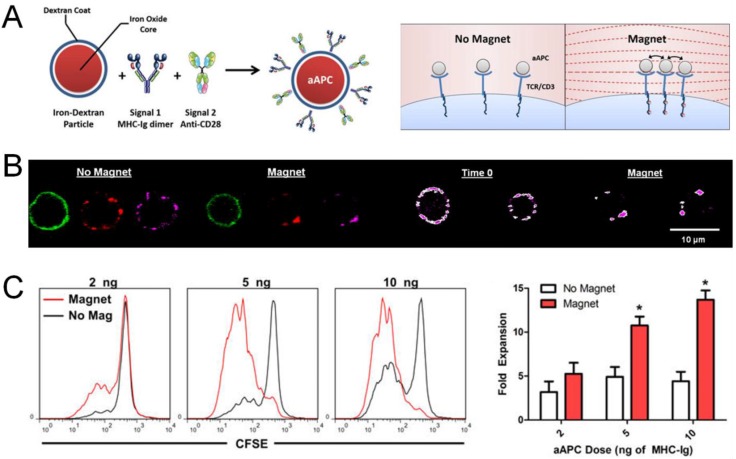
Artificial antigen-presenting cells (aAPCs) for activation of T cells. (**A**) aAPCs composed of an iron nanoparticle core and dextran shell conjugated with stimulatory molecules induced clustering of T-cell receptors (TCRs) under magnetic field; (**B**) TCR clustering was visualized by fluorescence imaging. Green, lymphocyte marker on T cells; red: aAPCs; magenta: CD3ε on T cells; (**C**) Proliferation of T cells was enhanced by aAPC-induced TCR clustering *in vitro*. Reproduced with permission [95].

There has been increasing interest in artificial APCs (aAPCs) surface-decorated with covalently conjugated tumor antigen/MHC complex and anti-CD28 antibody. The rationale is that direct activation of antigen-specific T cells by aAPCs will obviate the need for antigen delivery to APCs and antigen processing and presentation, while also avoiding potential activation of immune checkpoint molecules, such as CTLA-4, expressed on T cells via the use of agonist antibodies directed toward co-stimulatory pathways. Indeed, various particle platforms have been explored for aAPCs, including PLGA microparticles [93], liposomes [94], iron/dextran nanoparticles [95], and carbon nanotubes [96]. In particular, aAPCs composed of an iron nanoparticle core and stimulatory molecules on the dextran shell were shown to induce T-cell receptor clustering when incubated with T cells under magnetic field, thus allowing external stimulus-induced proliferation of antigen-specific T cells *in vitro* and *in vivo* (Figure 4) [95]. Moreover, carbon nanotubes loaded with activation signals have been also developed as aAPCs to expand antigen-specific CD8^+^ T cells, which were then successfully used for adoptive T cell therapy [96]. Interestingly, recent studies have revealed that ellipsoidal PLGA nano/microparticles were more efficient aAPCs than their spherical counterparts [97,98], demonstrating that biophysical parameters of aAPCs may play a crucial role in induction of T cell responses.

## 5. Synthetic Delivery Approaches to Induce Immunogenic Cell Death (ICD)

Recent studies have demonstrated immunogenicity of dying cancer cells under certain chemotherapies or radiotherapy [99]. Although systemic administration of chemotherapeutics is generally immunosuppressive, *in situ* treatments with certain chemodrugs especially anthracyclines, such as doxorubicin and mitoxantrone, have been shown to induce ICD [100,101]. In addition, the abscopal effect observed during radiotherapy, *i.e.*, regression of distant, non-irradiated tumors, is also believed to be caused by systemic immune responses elicited by dying primary tumor cells [102]. Since the initial discovery of ICD, anthracycline chemodrugs have been investigated widely for immune-mediated anti-tumor efficacy in addition to their direct tumor-killing effects, especially in combination with other cancer immunotherapies, such as vaccines, adoptive cell transfer, and checkpoint inhibitors [103]. Indeed, ICD suggests an alternative approach for whole-cell vaccination based on *ex vivo* generated immunogenically dying tumor cells or induction of ICD *in situ*. In addition, co-delivery of adjuvants with ICD inducers may be helpful to potentiate anti-tumor immune responses, thus motivating the use of adjuvant-carrying particulate delivery systems for further enhancing ICD. For example, PLGA microparticles have been employed to encapsulate doxorubicin and CpG and intratumorally injected for induction of ICD [104]. In addition, doxorubicin-based *in situ* vaccination strategy combined with anti-CTLA-4 and anti-OX40, an agonistic antibody against the stimulatory checkpoint molecule OX40, has been shown to improve T cell infiltration into distant tumors, leading to tumor eradication and increased survival [105].

## 6. Synthetic Delivery Systems Targeted to Immune Checkpoints

Cancer immunotherapy aiming to reverse immunosuppression has achieved striking success in recent years. The CTLA-4 inhibitory antibody Ipilimumab has improved the survival of patients with advanced, untreatable melanoma by 3.7 months, and gained FDA-approval as a new category of cancer immunotherapy [106]. However, treatment with Ipilimumab was also accompanied with adverse events and moderate response rates [5]. The PD-1 inhibitory antibodies Nivolumab and Pembrolizumab were also approved for the treatment of malignant melanoma in late 2014. PD-1 is now considered a better target than CTLA-4 because antibody-mediated blockade of PD-1 among tumor-infiltrating T cells within the tumor microenvironment leads to mitigated side effects and higher response rates [107], especially among patients with PD-L1 positive tumors [6,108]. In addition, dual inhibition of CTLA-4 and PD-1 recently has been shown to be more efficacious than a single therapy, mainly due to their distinctive mechanisms of action: the main sites of action for antibodies against CTLA-4 and PD-1 are thought to be within lymphoid tissues and tumor regions, respectively. However, the current systemic administration route for these therapeutic antibodies may still cause off-target toxicity. A previous study has addressed this issue by intratumoral administration of anti-CTLA-4 antibody with mesoporous silica [109]. The micron-size carrier with nanopores achieved high loading efficiency and enhanced anti-tumor efficacy, compared with soluble antibody injected intraperitoneally in a murine melanoma model, possibly due to controlled release of antibody from an *in situ* depot. Alternatively, targeted delivery of siRNA against PD-L1 has also been investigated with cationic lipoid and polymeric nanoparticles [110,111]. PD-L1 expressed on cancer cells were efficiently silenced by siRNA complexed with folic acid-modified polyethylenimine, resulting in enhanced *in vitro* T-cell activation [111].

In addition to immune checkpoint inhibitors covered above, there are also stimulatory checkpoint targets, such as OX40 (CD134) and 4-1BB (CD137), which can be activated to improve anti-tumor immunity. Both molecules belong to the receptor family of tumor necrosis factor (TNF) and directly induce T-cell activation. Ligation of OX40 on T cells with its ligand on APCs results in activation of both CD4^+^ and CD8^+^ T cells, leading to inhibition of tumor growth [112]. Notably, expansion of T_regs_ following activation of OX40 remains a controversial topic [113,114]. Since all CD4^+^ T cell subtypes can be activated by the OX40 pathway, it is likely that induction of T_regs_ depends on the particular polarizing cytokine milieus that the cells are exposed to. In contrast to OX40, 4-1BB preferentially activates CD8^+^ rather than CD4^+^ T cells [115]. 4-1BB is up-regulated as a surrogate for CD28 which cannot compete against CTLA-4 in binding to co-stimulatory molecules during the late or secondary immune response [116]. Nanoparticle delivery systems have been developed for these antibody therapeutics, aiming to mimic the natural immune activation by antibodies surface-displayed on particles or to reduce systemic toxicity by localized administration. Anti-OX40 antibody was conjugated to the surfaces of PLGA nanoparticles via EDC/NHS chemistry and promoted antigen-specific killing by CD8^+^ T cells *in vitro* [117]. In another combinational therapeutic approach, anti-4-1BB antibody and IL-2 were separately displayed on liposomal surfaces for localized tumor therapy, inducing robust anti-tumor CTL responses, while minimizing off-target side effects and preventing cytokine storm typically observed after systemic administration of immunotherapeutics [118].

## 7. Synthetic Delivery Systems for Adoptively Transferred T Cells

**Figure 5:**
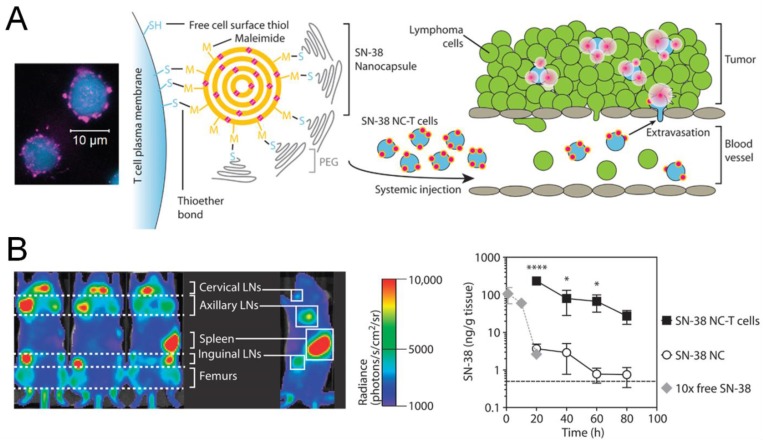
Engineered T cells for cancer therapy. (**A**) T cells (blue) conjugated with nanoparticles (magenta) loaded with a chemodrug (SN-38) were used for drug delivery to lymphoma; (**B**) T cells with surface-bound chemodrug preferentially accumulated in tumor-bearing lymphoid tissues following systemic administration, and significantly improved the drug distribution in lymph nodes, compared with equivalent or 10-fold higher dose of the nanoparticulate or soluble drug, respectively. Reproduced with permission [124].

Adoptive cell therapy (ACT), based on autologous T cells expanded with tumor antigens and IL-2 *ex vivo*, is envisioned to induce tumor regression as a “live drug”. However, this approach is limited by moderate responses, due to insufficient expansion of transferred T cells and inefficient trafficking to tumor regions [119], as well as potential severe side effects characterized by “cytokine storm” with TNF, IFN-γ and IL-6 when IL-2 is systemically co-administered with T cells [120]. To address these limitations, nanoparticle-based cellular engineering approaches have been examined to improve the therapeutic efficacy of ACT. Maleimide-modified synthetic nanoparticles were conjugated to the surfaces of CD8^+^ T cells via sulfhydryl groups exposed by cell-surface proteins [121]. T-cell stimulating cytokine complexes, IL-15/IL-15Ra and IL-21, were encapsulated into nanoparticles to provide signals for T cell expansion *in situ*. This strategy resulted in potent proliferation of transferred T cells and eradicated metastatic melanoma tumors in mice, whereas co-administration of T cells mixed with those cytokines failed to eliminate tumors. Tumor-specific T cells “equipped” with small-molecule inhibitors against T-cell exhaustion using the same method also showed therapeutic benefits [122]. In an alternative strategy, *ex vivo* expanded antigen-specific CD8^+^ T cells and T-cell activating particles were co-delivered via an implantable biodegradable hydrogel system to treat residual tumors as well as metastases in murine breast cancer models [123]. Within the local implant, lipid-coated mesoporous silica particles encapsulating IL-15 and IL-15Ra while displaying stimulatory antibodies against CD3, CD28, and CD137 efficiently activated T cells, which were gradually released from the depot and more efficacious than systemic or local administration of T cells without stimulatory signals. In addition to CTL-mediated tumor-specific killing, T cells also have been utilized to shuttle therapeutics to diseased tissues. As shown by a recent study on a metastatic murine lymphoma model, autologous polyclonal T cells with the potential to home to tumor-bearing lymphoid tissues were primed *ex vivo* and conjugated with chemodrug-loaded nanoparticles on the cell surfaces (Figure 5A) [124]. This “Trojan horse” approach exploiting innate tropism of T cells to lymphoid tissues allowed selective delivery of the chemotherapeutic to disseminated lymphoma tumors, with 90-fold greater concentration of the drug accumulated in lymph nodes than free drug systemically injected at 10-fold higher doses (Figure 5B). This T cell-mediated delivery significantly reduced tumor burden and improved survival, compared with administration of free drug or drug-loaded nanoparticles alone.

## 8. Conclusions

This review article has covered various applications of nanotechnology in cancer vaccination and immunotherapy. Therapeutic strategies based on nanomaterials can enhance the efficacy of cancer vaccines by improving lymphatic delivery of tumor antigens or by incorporating targeting approaches and/or stimuli-responsive materials to modulate immune activation. Moreover, adjuvants loaded into nanocarriers via hydrophobic or electrostatic interactions can further increase immunogenicity of tumor antigens. In addition, nanoparticulate drug delivery systems have been applied to augment the therapeutic potential of autologous DCs and synthetic artificial APCs, immunogenic cell death, immune checkpoint inhibitors, and adoptive T-cell therapy.

New horizons in cancer immunotherapy include identification of tumor neo-antigens by tumor exomes sequencing and utilization of neo-antigens for “precision medicine” by tailoring the cancer vaccine for each patient [125]. As mutated neo-antigens should be more immunogenic than self-antigens over-expressed on tumor cells, this approach may dramatically improve the therapeutic efficacy of cancer vaccines, as demonstrated by tantalizing results in recent studies [126,127]. However, these vaccination strategies based on neo-antigens are still subjected to delivery issues facing conventional subunit vaccination; therefore, vaccine nanoparticles loaded with neo-antigens may address these challenges and offer personalized nanomedicines designed to elicit anti-tumoral immune responses. In addition, combination therapies utilizing cancer vaccines and immune checkpoint inhibitors merit further exploration as they could simultaneously drive potent anti-tumor immune responses and reverse immunosuppression in tumor microenvironment. Indeed, recent remarkable clinical results observed after dual blockade of PD-1 and CTLA-4 have highlighted advantages of combinational immunotherapies with distinct targets or mechanisms. Furthermore, numerous nanocarriers designed for delivery of conventional chemotherapeutics can be potentially applied to initiate and enhance immunogenic cell death and may further provoke the immune system against tumor cells when co-administered with cancer immunotherapeutics. In conclusion, nanoparticle delivery systems are versatile platforms that can be rationally designed to address key technical challenges in cancer vaccination and immunotherapy and may offer cutting-edge material-based strategies to advance this exciting field of cancer therapy.

## Abbreviations

aAPC: Artificial antigen-presenting cell
ACT: Adoptive cell therapy
APCs: Antigen-presenting cells
CpG: Unmethylated oligonucleotide containing CpG motif
CTLA-4: Cytotoxic T-lymphocyte-associated protein 4
CTL: Cytotoxic T lymphocyte
DCs: Dendritic cells
EBV: Epstein-Barr virus
GM-CSF: Granulocyte macrophage colony-stimulating factor
HLA: Human leukocyte antigen
HPV: Human papilloma virus
ICD: Immunogenic cell death
LPS: Lipopolysaccharide
MDSCs: Myeloid-derived suppressor cells
MHC: Major histocompatibility complex
MPLA: Monophosphoryl lipid A
OVA: Ovalbumin
PD-1: Programmed death-1
PD-L1: Programmed death ligand-1
PLGA: Poly(lactic-co-glycolic acid)
siRNA: Small-interfering RNA
TCRs: T-cell receptors
TNF: Tumor necrosis factor
T_H_ cells: Helper T cells
TLRs: Toll-like receptors
TNF: Tumor necrosis factor
T_regs_: Regulatory T cells
Trp-2: Tyrosine-related protein 2
